# Marantic Endocarditis in Metastatic Lung Adenocarcinoma

**DOI:** 10.14797/mdcvj.1243

**Published:** 2023-08-01

**Authors:** Anirudh Palicherla, Suma Pusapati, Dixitha Anugula, Abhishek Thandra

**Affiliations:** 1 Creighton University School of Medicine, Omaha, Nebraska, US; 2 Houston Methodist, Houston, Texas, US

**Keywords:** marantic endocarditis, adenocarcinoma, transesophageal echocardiogram

## Abstract

Marantic endocarditis is a rare condition associated with autoimmune disease, malignancy, and hypercoagulable states. It is characterized by sterile friable vegetations composed of fibrin and platelets that confer a high risk of systemic embolism. Here we showcase imaging that led to the diagnosis of an interesting case of marantic endocarditis secondary to metastatic malignancy.

Marantic endocarditis is a rare noninfectious endocarditis characterized by the deposition of sterile platelet thrombi that can lead to systemic embolism. Also known as nonbacterial thrombotic endocarditis (NBTE), Libman-Sacks endocarditis, or verrucous endocarditis, marantic endocarditis is different from culture-negative endocarditis, which is due to infectious etiologies that are not readily identified.

A 71-year-old White male with a past medical history of atrial flutter, severe aortic stenosis with mild to moderate aortic regurgitation, and recently diagnosed stage IV metastatic lung adenocarcinoma presented to the emergency department. He reported shortness of breath and fatigue for 2 to 3 weeks. Transesophageal echocardiogram revealed abnormal thickening of aortic valve leaflet tips with moderate eccentric aortic regurgitation (Figure 1; Videos 1-5). Magnetic resonance imaging of the brain showed small diffusion positive infarcts involving cortex and subjacent white matter within multiple vascular distributions in both cerebral hemispheres, suggestive of a recent embolic event. Blood cultures were negative throughout his hospitalization.

**Figure 1:**
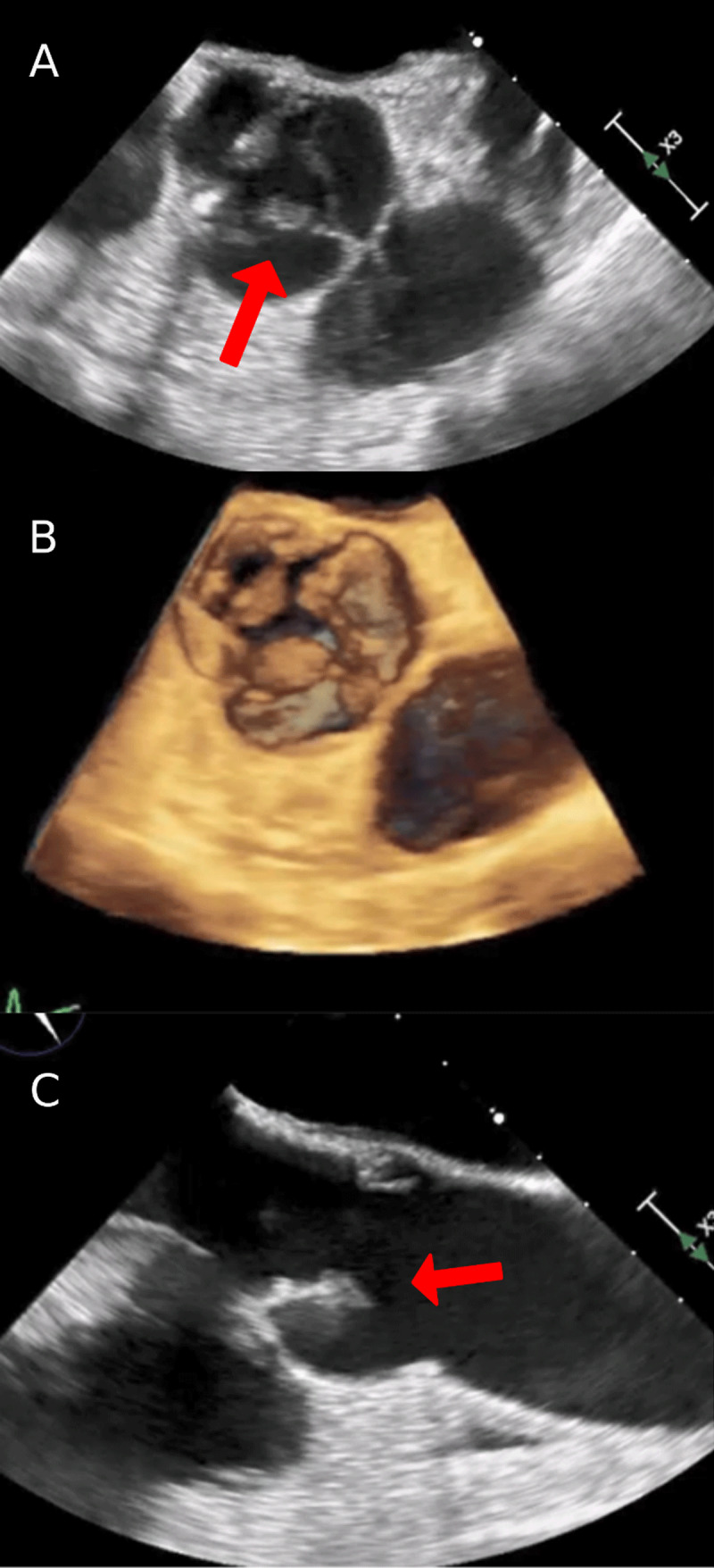
**(A)** Transesophageal echocardiogram (TEE) with short axis view of the aortic valve showing thickened non and right coronary cusp leaflet tips (arrow). **(B)** 3-dimensional image of TEE with short axis view of the aortic valve showing thickened non and right coronary cusp leaflet tips. **(C)** TEE with long axis view of the aortic valve showing thickened leaflet tips (arrow).

**Video 1:** Aortic valve short axis view on transesophageal echocardiogram showing thickened valve leaflets; see also at https://youtu.be/5X9KmH67t2M.

**Video 2:** Color comparison of short axis view with mild to moderate aortic regurgitation; see also at https://youtu.be/GClgeiMwmn4.

**Video 3:** 3-dimensional view of the aortic valve on transesophageal echocardiogram; see also at https://youtu.be/YOmj-ZTvCgY.

**Video 4:** Long axis view of the aortic valve with thickened leaflet tips; see also at https://youtu.be/PGglbOzrSxE.

**Video 5:** Color comparison of long axis view of the aortic valve showing aortic regurgitation; see also at https://youtu.be/FUrDwpdNdJQ.

Diagnosis required ruling out infection and establishing the presence of valvular vegetations using echocardiography. Vegetations are frequently left-sided with about 60% to 65% of cases involving the mitral valve, up to 25% involving the aortic valve, and, less commonly, involving both valves.^1,2^ NBTE vegetations are typically small (< 1 cm in diameter), broad based, and irregular in shape.^2^ Main complications are embolic, as lesions of marantic endocarditis are more friable and prone to systemic embolization, which occurs in up to 40% of patients.^3,4,5^
